# Editorial: Advancements in understanding zoonotic parasitic diseases

**DOI:** 10.3389/fvets.2024.1539556

**Published:** 2025-01-14

**Authors:** Rodrigo Morchón, Simona Gabrielli, Lavinia Ciuca, Ettore Napoli, Elena Carretón

**Affiliations:** ^1^Zoonotic Disease and One Health Group, Faculty of Pharmacy, Biomedical Research Institute of Salamanca (IBSAL), Centre for Environmental Studies and Rural Dynamization (CEADIR), University of Salamanca, Salamanca, Spain; ^2^Department of Public Health and Infectious Diseases, Sapienza University of Rome, Rome, Italy; ^3^Department of Veterinary Medicine and Animal Production, University of Naples Federico II, Naples, Italy; ^4^Department of Veterinary Sciences, University of Messina, Messina, Italy; ^5^Internal Medicine, Veterinary Medicine and Therapeutic Research Group, Faculty of Veterinary Medicine, Research Institute of Biomedical and Health Sciences (IUIBS), Universidad de Las Palmas de Gran Canaria, Las Palmas de Gran Canaria, Spain

**Keywords:** zoonosis, One Health, humans, animals, wildlife

The field of zoonotic parasitic diseases, which encompasses infections transmissible between animals and humans, is currently undergoing significant advances, underscoring their growing importance in global public health. These diseases, caused by diverse range of pathogens including protozoa, helminths and ectoparasites, exhibit a broad spectrum of symptoms and severities, some of which result in severe acute illness or even death. Transmission of these parasites can occur through a variety of sources, including contaminated food and water, direct contact with domestic and wild animals, and vector-borne pathways involving insects or other arthropods. Despite the critical impact of zoonotic parasitic diseases, their detection, treatment, and control are often hindered by incomplete understanding and fragmented knowledge of their complex life cycles, host interactions, and environmental reservoirs (1, 2).

These challenges are further exacerbated by environmental and societal factors. Global warming is altering the habitats and distribution of both vectors and hosts, leading to the emergence and re-emergence of zoonotic diseases in previously unaffected regions (3). Additionally, the globalization of trade and travel facilitates the rapid movement of animal and human reservoirs, introducing parasites and vectors into new geographic areas and increasing the risk of outbreaks. These factors call for a comprehensive and integrated approach to the management of zoonotic parasitic diseases (4).

The primary aim of this Research Topic is to enhance the understanding of zoonotic parasitic diseases, especially those that are emerging and that have recently come to widespread public attention. This includes deepening the knowledge of their biology, epidemiology, and the local, regional, national or global advances in their diagnosis, treatment and control. Additionally, the Research Topic aims to facilitate the exchange of updated information on these diseases, particularly in relation to their proteomics, immunology and molecular biology, as well as new vaccines and diagnostic tools.

This Research Topic has gathered 15 manuscripts−12 original research papers, one short research paper, one case report and one opinion paper—with contributions from 104 authors. These papers focus on zoonotic parasitic diseases and related areas, covering Research Topics such as the biology and epidemiology of these diseases; advances in their diagnosis, treatment and control; studies on parasite-host relationships; research in proteomics, immunology and molecular aspects; the development of new vaccines and diagnostic tools; and illustrative clinical cases of these conditions.

The team of Rondón et al. has contributed to the detection of *Iodamoeba bütschlii, Dientamoeba fragilis*-like*, Giardia* sp., *Balantidium/Buxtonella* sp., *Capillaria* sp., *Trichuris* sp., strongyliform larvae, and *Oesophagostomum* sp. potentially zoonotic parasites, in primates from a zoo in Italy. The results provide important and necessary information that justifies the generation of adequate safety measures for both visitors and animal keepers. Another study, by Cancino-Faure et al., searched for the presence of zoonotic filarial nematodes in mosquitoes [*Aedes* (*Ochlerotatus*) *albifasciatus* and *Culex pipiens*] and dogs in a previously unstudied semi-rural area of Central Chile, finding *Acanthocheilonema reconditum* and *Setaria equina*; although the authors did not detect the presence of zoonotic parasites, they did stress the importance of continuous surveillance, especially in areas that are not regularly monitored.

de Andrade Vieira et al. analyzed the presence of *Dirofilaria immitis* in an area of Rio de Janeiro (Brazil) showing the expansion of the disease and highlighting the importance of the use of prophylactic measures and awareness of both health personnel and dog owners to interrupt the spread and establishment of heartworm disease. On the other hand, Esteban-Mendoza et al., through the application of molecular and morphological characterization techniques, demonstrated the importance of their use in the detection of microfilaremic dogs infected by *D. immitis* and *A. reconditum*, and their usefulness in making an accurate diagnosis to establish an appropriate treatment for each filarial species. Likewise, and in relation to the study of diagnostic techniques, the study carried out by Albasyouni et al. underlines the need to use molecular techniques to describe intestinal coccidian parasites (*Eimeria* spp.) together with morphological tools in birds (pigeons).

The research of Liu et al. presented a study in which molecular techniques were applied to detect several zoonotic species of *Cryptosporidium* spp. and others adapted to wild rodents in a province of China. Similarly, Uakhit et al. identified *Baylisascaris* spp. by molecular and phylogenetic analyses in wild carnivores from different regions of Kazakhstan, highlighting their potential risk of infection to humans. These findings underscore the importance of a multidisciplinary “One Health” approach to prevent the spread of such pathogens.

Also, Bandelj et al. presented, for the first time, a case report describing the presence of *Gongylonema pulchrum*, a potentially zoonotic parasite, in a Slovenian roe deer using morphological and molecular techniques. Moreover, Elshahawy et al. conducted a study to molecularly characterize leukocytozoonosis in pigeons in Egypt, with the aim of developing more effective control and prevention strategies to limit the spread of infection to other birds.

Rodríguez-Escolar, Balmori-de la Puente et al. and Rodríguez-Escolar, Hernández-Lambraño et al. conducted two studies focused on controlling vector-borne zoonotic diseases, developing infection risk maps for *Dirofilaria* spp. in Serbia and canine leishmaniasis in Spain and Portugal. To this aim, they took into account the habitat suitability map for their main vectors, the weighting of these maps with the parasite behavior in these vectors and their validation with the geolocation of infected dogs. This approach offers high predictive accuracy, providing an excellent tool for the control and prevention of these diseases.

In addition, Raw et al. highlight the relevance of conducting efficient and effective deworming programs in dogs for *Ancylostoma caninum* that can be administered regularly without the need for veterinary supervision in Australian Aboriginal communities where veterinary visits may be limited. In reference to leishmaniasis, Cavalera et al. report that the seasonality of anti-*L. infantum* titres in dogs should be considered in the design of clinical trials to evaluate treatments and preventive measures for canine leishmaniasis, which would enhance the efficacy of control strategies.

In relation to parasite-host relationships and the resulting immune response, Chai et al. presented data on the T cell-mediated immune response in the maintenance of intestinal immune homeostasis and the impact of *Moniezia benedeni* infections in sheep, particularly in altering immune cell densities. In addition, the role of a recombinant protein (rEg.P29) from *Echinococcus granulosus* as a potential epitope peptide vaccine is explored by Yang et al., emphasizing its relevance given the zoonotic significance of this parasite.

We would like to express all our gratitude to all 104 researchers who have contributed to this Research Topic by sharing their valuable studies on zoonotic parasitoses from the “One Health” perspective. We also extend our thanks to the reviewers and staff of *Frontiers in Veterinary Science*, whose efforts have ensured the successful completion of this Research Topic.
